# Retrospective screening reveals the rare occurrence of zoonotic *Strongyloides stercoralis* in dogs from temperate Australia, 2014–2024

**DOI:** 10.1017/S0031182026101693

**Published:** 2026-04

**Authors:** Jaisy Chong, Jan Šlapeta, Maira Nascimento Meggiolaro, Jennifer Green, Rogan Lee, Michael P. Ward, Nicolle Kirkwood

**Affiliations:** 1 Sydney School of Veterinary Science, Faculty of Science, The University of Sydney, Sydney, NSW, Australia; 2 Sydney Infectious Diseases Institute, The University of Sydney, Sydney, NSW, Australia; 3 New South Wales Health Pathology, Centre for Infectious Diseases and Microbiology Laboratory Services, Level 3 Institute of Clinical Pathology and Medical Research (ICPMR), Westmead, NSW, Australia; 4 School of Biomedical Sciences, Faculty of Medicine and Health, The University of Sydney, Sydney, NSW, Australia

**Keywords:** neglected soil transmitted helminth, quantitative PCR, *Strongyloides stercoralis*, strongyloidiasis, temperate region, threadworm

## Abstract

*Strongyloides stercoralis* is an intestinal nematode capable of infecting humans and dogs. Occurrence in dogs from temperate, traditionally non-endemic regions remains poorly characterized, often due to the absence of accessible veterinary diagnostic tests. Recent reports of infections from temperate metropolitan areas have raised concerns about the extent of *S. stercoralis* prevalence in dogs in these regions. This study investigated the presence of *S. stercoralis* DNA in canine faecal samples from Sydney, New South Wales, Australia, using a real-time quantitative PCR (qPCR) targeting the 18S rRNA gene, with a limit of detection of 2 DNA copies. Archived faecal DNA (*n* = 448) collected between 2014 and 2024 from 2 university veterinary hospitals were screened. Of all samples, 1 (0.02%) was positive for *S. stercoralis* DNA, corresponding to approximately 6.8 × 10^3^ 18S rDNA copies, equivalent to 3.2 of *S. ratti* third-stage larvae, per 250 mg of dog faeces. Partial *cox1* and 18S rDNA loci (HVR-I and HVR-IV) deep amplicon sequencing confirmed that *S. stercoralis* circulates between dogs and humans. The positive sample originated from a Border Collie puppy presenting with gastrointestinal signs. Although detection was rare, this confirms the parasite’s presence in companion dogs within a temperate urban environment. The results highlight the diagnostic utility of 18S rDNA-based qPCR for retrospective surveillance and support the inclusion of *S. stercoralis* in molecular diagnostic panels for dogs with gastrointestinal disease. Expanded, targeted molecular and coproscopic surveillance is warranted to clarify the prevalence, distribution and zoonotic potential of *S. stercoralis* in dogs across Australia’s non-endemic regions.

## Graphical abstract

**Figure:**
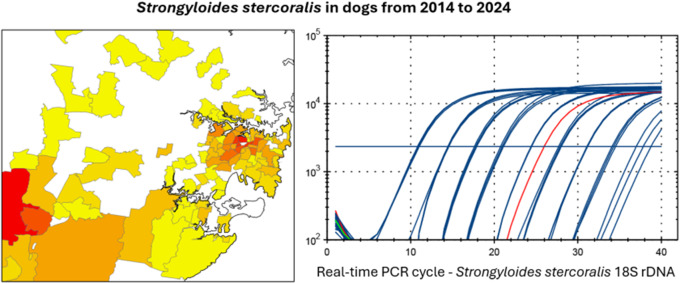

## Introduction

*Strongyloides stercoralis* is a soil-transmitted helminth capable of infecting both humans and dogs, with a global distribution primarily concentrated in tropical and subtropical regions (Schnyder et al., 2022; Buonfrate et al., 2023; Colella et al., 2024; Zhao et al., 2025). Despite its zoonotic potential and an estimated global burden exceeding 600 million human infections, the parasite’s presence in dogs has been historically under-recognized (Czeresnia and Weiss, 2022; Eslahi et al., 2022; Buonfrate et al., 2023). Recent case reports from temperate metropolitan areas, including south-eastern Australia, Austria, Czech Republic, Finland, Italy, Romania, Switzerland, United Kingdom and United States, have highlighted emerging canine infections and raised concerns about the true extent of *S. stercoralis* prevalence in temperate regions, where the parasite is not traditionally considered endemic (Dillard et al., 2007; Snook et al., 2009; Zanzani et al., 2014; Graham et al., 2019; Schnyder et al., 2022; Unterköfler et al., 2022; Borrás et al., 2023; Cagnasso et al., 2023; Chapman et al., 2024; Deak et al., 2024; Kirkwood and Šlapeta, 2024; Nosková et al., 2024; Chen et al., 2025; Mace and Barker, 2025). The apparent underestimation of canine infections is likely due to several factors, including the lack of reliable and routinely used diagnostic tools, asymptomatic carriers escaping detection and limited awareness among veterinarians (Olsen et al., 2009; Paradies et al., 2017; Buonfrate et al., 2022; Colella et al., 2024). Currently, no widely accessible reference diagnostic test exists for dogs, and available methods suffer from variable sensitivity and specificity due to low and intermittent larval shedding (Verweij et al., 2009; Buonfrate et al., 2018).

Molecular studies have identified 2 genetically distinct clades of *S. stercoralis* found in dogs, one found exclusively in dogs and the other shared with humans, indicating the parasite’s zoonotic potential (Jaleta et al., 2017; Nagayasu et al., 2017; Zhou et al., 2019; Bradbury et al., 2021). Infection with either clade can be self-limiting; however, immunocompromised dogs may exhibit non-specific gastrointestinal and respiratory signs that are frequently misattributed to more common conditions such as bacterial enteritis, *Giardia* infections or parasitic pneumonia attributed to angiostrongylosis (Chapman et al., 2024; Colella et al., 2024; Chen et al., 2025). In severe cases, humans can develop hyperinfection syndrome, a rapidly progressing and often fatal condition with a case fatality rate exceeding 60%, even with anthelmintic treatment, due to overwhelming worm burdens and systemic dissemination (Czeresnia and Weiss, 2022; Lo et al., 2025). Despite its clinical significance, the prevalence of *S. stercoralis* occurrence in dogs and the associated risk of zoonotic transmission in temperate regions remain poorly understood (Jaleta et al., 2017; Basso et al., 2019; Kirkwood and Šlapeta, 2024; Mace and Barker, 2025).

This study investigates the potential emergence of *S. stercoralis* infections in dogs in Sydney, a temperate region not traditionally considered endemic. Archival frozen dog faecal DNA samples collected between 2014 and 2024 were screened using real-time quantitative PCR (qPCR). Samples were sourced from the Veterinary Pathology Diagnostic Services (VPDS), The University of Sydney, which stored submissions from the University Veterinary Teaching Hospitals in Sydney (UVTHS) and Camden (UVTHC).

## Materials and methods

### Canine faecal DNA samples

Archived frozen canine faecal DNA samples (estimated to have undergone fewer than 10 freeze-thaw cycles) were retrieved from the VPDS biobank. Samples were collected between 2014 and 2024 from 2 University of Sydney veterinary hospitals, the UVTHS (postcode 2050) and the UVTHC (postcode 2570). These samples were originally submitted as part of routine diagnostic investigations for dogs presenting with various gastrointestinal signs and tested using the Small Animal Diarrhoea panel, a multiplexed-tandem real-time PCR assay and system (AusDiagnostics, Australia) (e.g. Meggiolaro et al., 2019).

In accordance with VPDS protocols, DNA extraction was performed on the day of sample receipt or the following day. DNA was isolated from 250 mg of faeces using 1 of 3 commercial kits: ISOLATE II Fecal DNA Kit (Bioline, Australia), PowerSoil DNA Isolation Kit (MoBio, Australia) or MagMax CORE (Thermo Fisher Scientific, Australia). Following diagnostic testing, DNA samples were stored at −20 °C for short-term use and subsequently transferred to −80 °C for long-term archival.

Data about each sample was collated in a spreadsheet including its clinical pathology (CP) number, host species (=dog), hospital origin (UVTHS, UVTHC) and year of collection. Individual dogs were identified by their hospital ID and for each dog a postcode was manually extracted from hospital medical records. To ensure data accuracy, we performed random sampling using Microsoft Excel v2509 (Microsoft Corporation); for each record, a random number was generated with the =RAND() function. The dataset was then sorted by these random values in ascending order, and the 30 records with the lowest values were selected. Postcodes for these records were manually cross-checked against the original source to confirm correctness.

### *Strongyloides ratti* larvae

An aliquot of an experimental culture containing around ∼1000 live *Strongyloides ratti* stage 3 larvae (L3) suspended in water was provided by the NSW Health Pathology Westmead Parasitology Unit (Sultana et al., 2013). Larvae were micromanipulated into individual sterile 1.5 mL Eppendorf tubes either with 1 larva or 5 larvae. DNA was extracted using the Monarch^®^ Spin gDNA Extraction Kit (New England Biolabs, Australis) following the animal tissue protocol, the DNA was eluted in 100 µL and stored in sterile 1.5 mL Eppendorf tubes at −20 °C prior use.

### Synthetic DNA *Strongyloides stercoralis*

This study used the synthetic *S. stercoralis* 18S rRNA gene fragment (AF279916). The synthetic DNA was obtained as gBlocks™ Gene Fragments (Integrated DNA Technologies, Australia). The synthetic DNA was resuspended in distilled water and a stock solution of 10^9^ copies/μL was stored in sterile 1.5 mL Eppendorf tubes at −20 °C prior use.

### Diagnostic qPCR for *Strongyloides stercoralis* and bacteria

Two probe-based TaqMan qPCR assays were used throughout this study (Table 1). The *S. stercoralis* qPCR assay was adopted from a previously developed method targeting 18S rRNA gene which was reported with 100% specificity and high to moderate sensitivity (Verweij et al., 2009; Sultana et al., 2013). Validation in humans has resulted in 93–95% specificity and variable sensitivity between 56.5% and 71.8% depending on the reference test used, infection intensity and sampling strategy (Buonfrate et al., 2018). There is a close evolutionary relationship and conserved gene sequences between *S. stercoralis* and *S. ratti*, resulting in cross-reactivity in PCR assays and *S. ratti* is generally used as a positive control (Verweij et al., 2009; Sultana et al., 2013; Cole et al., 2023). In addition, a bacterial load in each faecal sample was determined using a universal bacterial qPCR assay targeting the 16S rRNA gene, designed to amplify most of the groups of bacteria (Nadkarni et al., 2002).

**Table 1.** Summary of amplification primers used in this study Table 1 long description.

*Strongyloides stercoralis* 18S rRNA gene assay (101 bp)	
S1221_Stro18S-1530F	5′-GAA TTC CAA GTA AAC GTA AGT CAT TAG C-3′
S1222_Stro18S-1630R	5′-TGC CTC TGG ATA TTG CTC AGT TC-3′
S1220_Stro18S-2586T	5′ 6-FAM-ACA CAC CGG /ZEN/ CCG TCG CTG C-3′ IABkFQ

Bacterial 16S rRNA gene assay (∼450 bp)	
S1217_Bac-F	5′-TCC TAC GGG AGG CAG CAG T-3′
S1218_Bac-R	5′-GGA CTA CCA GGG TAT CTA ATC CTG TT-3′
S1219_Bac-P	5′ SUN-CGT ATT ACC /ZEN/ GCG GCT GCT GGC AC-3′ IABkFQ

Note: IABkFQ = Iowa Black Fluorescent Quencher.

All probes and primers were obtained from Integrated DNA Technologies (IDT, Australia). The *S. stercoralis* and bacterial probes were 5′-end labelled with FAM and SUN fluorophore, respectively, with an internal ZEN™ Quencher and 3′-end Iowa Black™ Fluorescent Quencher (Table 1). The Myra Liquid Handling System (Bio Molecular Systems, Australia) was used to prepare batches of qPCR reactions. All qPCRs were run using the CFX Opus 96 Real-Time PCR System (BioRad, Australia) with runs analysed using CFX Maestro 3.2 (BioRad, Australia) and cycle threshold (Ct) auto-calculated. All DNA template samples were centrifuged for 2 min (>10 000 × ***g***) using the Eppendorf 5424 Centrifuge (Eppendorf, Australia) before qPCR set-up.

The *S. stercoralis* qPCRs were run in 20 μL volume with 2 μL of source template, 10 μL of Luna^®^ Universal Probe qPCR Master Mix (New England Biolabs, Australia), 400 nM of forward and reverse primers and 200 nM of the probe. The cycling conditions for the runs were 95 °C for 1 min as initial denaturation followed by 40 cycles of 95 °C for 15 s and 60 °C for 30 s. Each batch of samples included triplicate *S. ratti* positive controls with 1 larva and 5 larvae, and 2 negative controls (water). A *S. stercoralis* qPCR positive sample was run twice. Serial dilutions of the *S. stercoralis* synthetic DNA, 2 × 10^8^ to 2 synthetic DNA template copy (2 µL) per reaction (20 μL), were run in triplicates to construct standard curves for the limit of detection (LOD) which was considered as the lowest concentration of DNA (copy numbers) detected in all 3 replicates.

The bacteria qPCRs were run in 10 μL volume with 1 μL of source template, 5 μL of Luna^®^ Universal Probe qPCR Master Mix (New England Biolabs, Australia), 400 nM of forward and reverse primers and 200 nM of the probe. The cycling conditions for the runs were 95 °C for 1 min as initial denaturation followed by 35 cycles of 95 °C for 15 s and 60 °C for 30 s. Each batch of samples included 2 negative controls (water). Serial dilutions of the *S. stercoralis* synthetic DNA, 2 × 10^8^ to 2 synthetic DNA template copy per qPCR reaction.

### Deep amplicon sequencing to characterize *Strongyloides* spp

The nuclear 18S rRNA gene regions (HVR-I and HVR-IV) and mitochondrial *cox1* loci were targeted by PCR to further type the material and assess its zoonotic potential (Bradbury et al., 2021). The PCR and analysis was performed as described previously (Kirkwood and Šlapeta, 2024). Briefly, all PCR reactions were performed in a total volume of 30 μL using the SensiFAST SYBR^®^ No-ROX Kit (Bioline, Australia) with 3 μL of DNA template. Primers were adapted for Illumina sequencing. Amplification was carried out on a CFX Opus 96 Real-Time PCR System (Bio-Rad, Australia) under the following cycling conditions: initial denaturation at 95 °C for 3 min, followed by 32 cycles of 95 °C for 5 s, 63 °C for 15 s and 72 °C for 15 s. A melt curve analysis was performed at the end of the run to confirm a single peak corresponding to a specific amplicon. Each PCR run included a no-template control (ddH_2_O) to monitor for contamination. Amplicons were submitted for deep sequencing using Illumina NGS at the Ramaciotti Centre for Genomics (University of New South Wales, Sydney, Australia) and sequenced on a MiSeq i100 platform (300 bp paired-end, Illumina). Raw FastQ files were processed in R v4.4.2. using package ‘dada2’ v1.34.0 to infer amplicon sequence variants (ASVs) and their abundance per amplicon (Callahan et al., 2016). ASVs represented by fewer than 10 sequences were removed as spurious. Species and haplotype/genotype assignment for *Strongyloides* was performed in CLC Main Workbench (v25.0, Qiagen, Australia) using blastn searches against the NCBI ‘nr’ database and a curated local reference set.

### Data analysis

All data analysis and associated figures were produced in R v4.4.2 using packages ‘dplyr’ v1.1.4, ‘readxl’ v1.4.5, ‘car’ v3.1-3, ‘ggplot2’ v4.0.1, ‘gridExtra’ v2.3 and ‘svglite’ v. 2.2.2. All Ct values were analysed to assess the effect of year and hospital. Normality of residuals was tested using the Shapiro–Wilk test and visualized with Q-Q plot and indicated violations to normality. A non-parametric approach was adopted using the Kruskal–Wallis test to evaluate the effect of year and hospital on Ct values. When significant differences were detected (*p* < 0.05), pairwise Wilcoxon rank-sum tests with Benjamini–Hochberg correction were performed for post hoc comparisons. The mean of all Ct values and 3 standard deviations were calculated and used as the upper threshold suggestive of possible DNA degradation or inhibition.

Sampling maps were created using ArcGIS Pro v3.1 (ERSI Inc., 2023). Number of samples were counted per postcode (Microsoft Excel) and postcode totals were imported and joined to a shapefile of New South Wales postcodes (Geocentric Datum of Australia 1994). Proportional symbol and choropleth maps were created based on samples per postcode. Distributions of samples per postcode were summarized by calculating mean centres and directional ellipses (+1 standard deviation, SD) (Spatial Analyst. ArcGIS Pro v3.1), for samples submitted to Sydney (UVTHS) and Camden (UVTHC) hospitals, separately. Finally, the clustering of number of samples per postcode was investigated using Moran’s autocorrelation index (Spatial Analyst. ArcGIS Pro v3.1).

## Results

### Confirmation of archival canine faecal DNA integrity using bacterial DNA amplification

**Figure 1.**
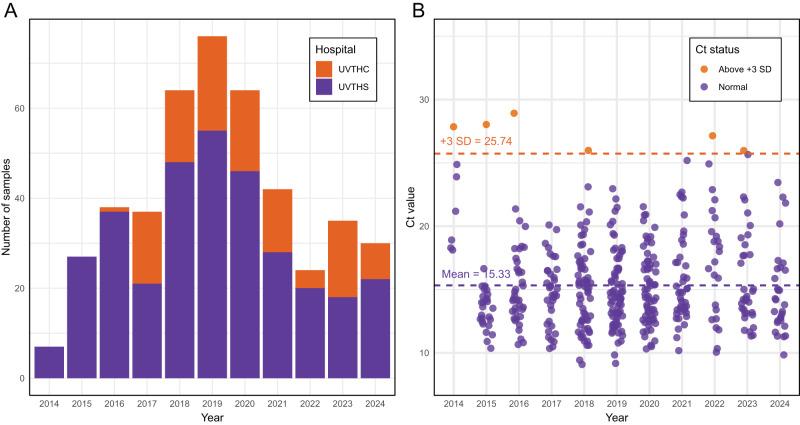
Archival canine faecal sample from 2014 to 2024. (A) Number of dog DNA samples (*n* = 448) obtained from the Veterinary Pathology Diagnostic Services (VPDS) archival storage from 2 hospitals, University Veterinary Teaching Hospital Sydney (UVTHS, *n* = 333) and University Veterinary Teaching Hospital Camden (UVTHC, *n* = 115). (B) Ct values of bacteria qPCR assay (*n* = 444) from 2014 to 2024. Dash line represents mean (Ct = 15.32) and the mean +3 standard deviation (SD) threshold (Ct = 25.71). Four samples did not produce Ct value (>35). Figure 1 long description.

Archived canine faecal DNA samples (*n* = 448), originally isolated over a 11-year period (2014–2024), were successfully retrieved for molecular analysis (Figure 1A). A total of 448 samples from 2 hospitals were included, comprising 333 from UVTHS and 115 from UVTHC. Before testing for *S. stercoralis* DNA, DNA integrity was assessed using a universal bacterial 16S rRNA gene assay to ensure sample quality had not degraded due to long-term storage or repeated freeze-thaw cycles.

Individual Ct values using the universal bacterial 16S rRNA gene assay were obtained for most samples (99%, 444/448), confirming amplifiable bacterial DNA. Four samples failed to yield Ct values (>35). The mean and standard deviation for Ct value across all samples was 15.33 ± 3.47 (95% CI: 15.01–15.65). A total of 6 out of 444 samples had Ct values above 25.7, corresponding to the mean plus 3 standard deviations, indicating possible DNA degradation or inhibition (Figure 1B). The Kruskal–Wallis test was used to evaluate the effect of year and hospital on Ct values (year: *c*^2^ = 28.205, *df* = 10, *p* < 0.05; hospital: *c*^2^ = 0.067, *df* = 1, *p* = 0.80). Pairwise Wilcoxon rank-sum tests with Benjamini–Hochberg correction revealed significant differences between 2014 and all subsequent years (*p* < 0.05). Additionally, 2015 differed significantly from 2022 (*p* = 0.048). No other year pairs showed significant differences (*p* > 0.05). After excluding 2014 and 2015, Kruskal–Wallis tests indicated no significant differences in Ct values among the remaining years (*p* > 0.05).

### Ensuring assay performance using *Strongyloides ratti* larvae as positive controls

Third-stage *S. ratti* larvae (L3) were included as positive controls in each qPCR run to assess assay consistency and performance. Two quantities, 1 larva and 5 larvae, were included in each run at triplicates. The mean and standard deviation of Ct value for a single larva was 28.37 ± 0.53 (95% CI: 28.15–29.59, *n* = 24), while 5 larvae produced a mean Ct value and standard deviation of 25.34 ± 0.45 (95% CI: 25.15–25.53, *n* = 24). These results demonstrated the assay’s high reproducibility across repeated tests.

### Detection of *Strongyloides stercoralis* and assay LOD

Of the 448 archived canine faecal DNA samples tested, 1 sample (1/448; 0.02%) was positive for *S. stercoralis* DNA by qPCR. The *Strongyloides-*positive canine sample presented to the UVTHC in 2020 (CP 20-10005) had a Ct value of 25.87 and 26.23 in a repeated qPCR, therefore, within 0.5 Ct value of the original qPCR run.

**Figure 2.**
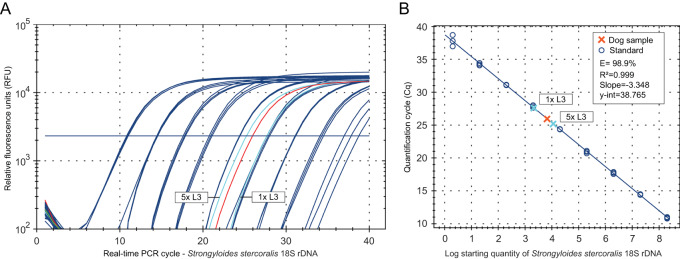
*Strongyloides stercoralis* qPCR assay for canine faecal sample DNA. (A) A triplicate amplification of qPCR using a synthetic *S. stercoralis* 18S rRNA gene (18S rDNA) with 9 serial dilutions from 2 × 10^8^ to 2 DNA template copy. The horizontal line represents the threshold for Ct values. A positive control standard samples from *Strongyloides ratti* single stage 3 larva (L3) and 5 L3 is shown and labelled (1 × L3, 5 × L3). The *Strongyloides*-positive dog faecal DNA sample is shown as red amplification curve. (B) Standard curve reconstructed from serial dilution of synthetic *S. stercoralis* 18S rRNA gene (18S rDNA). Empty circles show quantification of *S. ratti* 1 and 5 L3. The cross shows the *Strongyloides*-positive dog faecal DNA sample. Figure 2 long description.

Based on the standard curve of the synthetic *S. stercoralis* 18S rDNA serial dilution, a single *S. ratti* third-stage larva (L3) was equivalent to 2149 18S rDNA copies in the qPCR reaction, an equivalent to 1.1 × 10^5^ 18S rDNA copies in a single L3 (Figure 2A). Five larvae corresponded to 11 280 DNA copies (5.6 × 10^5^ 18S rDNA copies in 5 L3). The ratio of 18S rDNA copies between 1 L3 and 5 L3s was 5.24 (11 280/2149). The standard curve, generated from serial dilutions of synthetic *S. stercoralis* DNA, demonstrated high amplification efficiency (*E* = 98.9%) and strong linearity (*R*^2^ = 0.999), indicating excellent assay performance (Figure 2B). The LOD was determined to be 2 18S rDNA copies (in the qPCR reaction), consistently detected across all 3 replicates.

The *Strongyloides*-positive canine sample, with a Ct value of 25.87, corresponded to an estimated 6785 DNA copies and equivalent to 3.2 third-stage *S. ratti* larvae in the ∼250 mg of faeces (∼12.6 L3 g^−1^ of faeces) (Figure 2).

To further characterize and confirm the identity of the single *Strongyloides*-positive sample, we targeted partial *cox1* and 18S rDNA loci (HVR-I and HVR-IV). The DNA yielded Ct values of 29.26, 25.56 and 26.44 for *cox1*, HVR-I and HVR-IV, respectively. Deep Illumina amplicon sequencing yielded 17 186, 8104 and 25 355 high-quality sequences for *cox1*, HVR-I and HVR-IV, respectively. The *cox1* sequences were processed into a single ASV of 217 nt, which was >99% identical (216/217 nt) to *S. stercoralis* previously reported from a dog in Sydney. Phylogenetic placement confirmed the ASV as *S. stercoralis* within the cluster that includes isolates infecting both dogs and humans, ‘*cox1* lineage A’ sensu Bradbury et al. (2021). Using HVR-I, the single ASV (434 nt) was a perfect match to *S. stercoralis* haplotype VI, reported from dogs, humans and chimpanzees, as well as the Sydney dog case. Similarly, the HVR-IV amplicon (289 nt) yielded a single ASV matching haplotype A, also found in dogs, humans and chimpanzees, and identical to the sequence recovered from the Sydney case.

### Case history of the *S. stercoralis* positive 2-month-old Border Collie

The *S. stercoralis* DNA positive sample (CP 20-10005) was collected in December 2020. A 2-month-old Border Collie, domiciled at postcode 2570, and recently acquired from a breeder in NSW, presented to UVTHC with persistent diarrhoea, pasty faeces and haematochezia. Initial PCR (Small Animal Diarrhoea panel, AusDiagnostics) detected *Giardia intestinalis*, canine coronavirus, *Campylobacter* spp. and *Clostridium perfringens* enterotoxin; canine distemper virus was attributed to recent vaccination. The dog was treated with fenbendazole (50 mg/kg SID × 3 days) and metronidazole (15 mg/kg BID × 7 days) without resolution. At 5 months of age, diarrhoea persisted; repeat PCR on faecal DNA (CP21-01993) showed *G. intestinalis* and *C. perfringens* enterotoxin and fenbendazole was prescribed for 5 days. At 5 months of age, the faecal DNA (CP21-01993) was *Strongyloides-*negative. Clinical signs improved over the subsequent 3 months but recurred at 8 months of age; probiotics were initiated. At 13 months of age, diarrhoea recurred; PCR (Vetnostics; Canine multiplex vet faecal PCR, Laverty Pathology) detected *Giardia* and *Campylobacter jejuni*, and the dog was treated with fenbendazole for 5 days and metronidazole for 7 days. At 14 months of age, PCR was negative for all targets (Vetnostics). Faecal DNA from the 13- and 14-month samples was not available for retesting using *Strongyloides* qPCR. The dog remained on probiotics with occasional diarrhoea and no further antiparasitic treatment; it was healthy at 4.5 years of age.

### Dog sampling across Greater Sydney using archival samples

**Figure 3.**
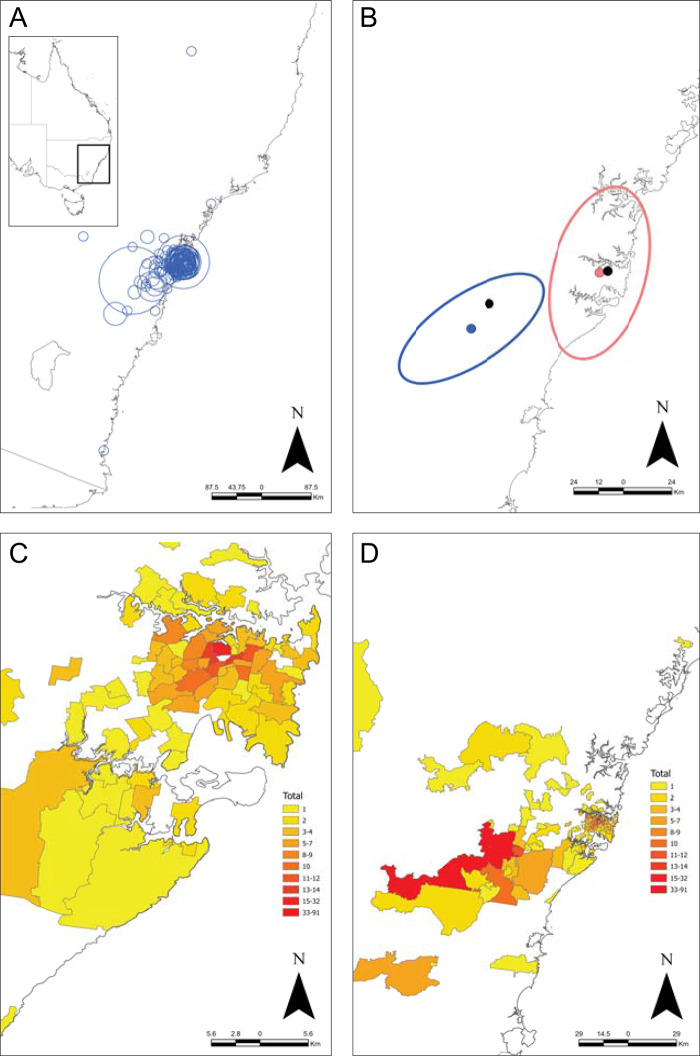
Canine faecal archival samples across Greater Sydney, NSW. (A) Proportional symbol maps of samples per postcode. The inset shows the region of Australia included in this study. (B) Mean centres and directional ellipses of the distributions of samples submitted to Sydney (UVTHS, red) and Camden (UVTHC, blue) hospitals. The locations of these 2 hospitals are shown as point dots (black). (C) Choropleth map of the number of samples per postcode submitted to Sydney (UVTHS) hospital. (D) Choropleth map of the number of samples per postcode submitted to Camden (UVTHC) hospital. Figure 3 long description.

We assessed the spatial distribution of dog sampling across Greater Sydney using archival DNA samples obtained from 2 veterinary hospitals. DNA records were linked to individual patients, and samples lacking postcode information were excluded (*n* = 11). In total, 408 individual dogs were included (UVTHS: *n* = 300; UVTHC: *n* = 108), all originating from New South Wales, Australia, with the majority located within the Greater Sydney region (Figure 3A). Mean centres and directional ellipses of sample distributions corresponded closely to the locations of the 2 hospitals in Sydney (UVTHS) and Camden (UVTHC) (Figure 3B). Spatial autocorrelation analysis revealed moderate clustering of samples in Sydney (UVTHS) (Moran’s *I* = 0.3150, *Z* = 11.5005, *p* < 0.0001) (Figure 3C), whereas no substantial clustering was observed in Camden (UVTHC) (Moran’s *I* = 0.0502, *Z* = 4.0222, *p* < 0.0001) (Figure 3D). Overall, sample distribution appeared to represent the source populations of the 2 hospitals included in this study.

## Discussion

In this retrospective study, we investigated the presence of *Strongyloides stercoralis* in dogs from a temperate climate zone using frozen archival faecal DNA samples collected over an 11-year period. The investigation was prompted by a clinical case of *S. stercoralis* diagnosed in February 2023 at UVTHS (Kirkwood and Šlapeta, 2024). This case involved an 8-month-old female French Bulldog presenting with a 1-week history of diarrhoea and a 24-h history of vomiting and lethargy (Kirkwood and Šlapeta, 2024). In response, we sought to determine whether *S. stercoralis* might be ‘*slipping under the diagnostic radar*’ of veterinary clinicians in temperate climate zones.

Across the 11-year period, 448 archived samples were tested, and only 1 was positive for *Strongyloides* spp. DNA using the highly sensitive assay that is commonly used in medical diagnostic laboratories (Verweij et al., 2009; Sultana et al., 2013). Using previously developed deep amplicon sequencing methods (Barratt et al., 2019; Beknazarova et al., 2019), we confirmed the presence of *S. stercoralis* and show that it belongs to the lineage infecting both dogs and humans. The detection of zoonotic 18S rDNA haplotypes, HVR-IV haplotype A and HVR-I haplotype VI, identical to those found in the Kirkwood and Šlapeta (2024) case was expected, as these haplotypes have been reported in both dogs and humans in Australia and are among the most commonly detected sequence types globally (Bradbury et al., 2021; Zhao et al., 2025).

It is unclear in the case of this 2020 positive case, the role that *S. stercoralis* played in the clinical presentation of the patient, as other potential co-pathogens, notably *G. intestinalis*, were detected in the first and subsequent faecal PCR testing and given that *S. stercoralis* can be detected in asymptomatic carriers (Buonfrate et al., 2022). Food intolerance or allergy was also considered a contributing factor. Acquisition of infection was likely to have occurred at the site of the breeder, located in NSW considering the limited time in the owner’s possession. Notably, at 5 months of age, there was no *S. stercoralis* DNA detected in this patient. Whilst the infection may have been self-limiting it is also possible that the treatment with fenbendazole for 3 days may have assisted in resolution of the infection (Thamsborg et al., 2017). Large-scale studies on the response to treatment of infected dogs are lacking and there are no registered products available for the treatment of *S. stercoralis* in dogs in Australia. Fenbendazole either used alone or in combination with moxidectin eliminated infections in some dogs, so it is feasible that this treatment may have helped clear the infection in this patient (Eydal and Skírnisson, 2016; Paradies et al., 2017). Off-label use of ivermectin, however, appears to be a more successful treatment with a variety of dose rates, frequencies and routes of administration described, however cannot be recommended in the absence of MDR-1 gene testing (Paradies et al., 2019; Chapman et al., 2024).

Although detection of *S. stercoralis* in dog faecal samples was rare, the finding confirms that *S. stercoralis* exists within temperate urban settings. This observation is consistent with recent Australian national surveillance data reporting *Strongyloides* spp. DNA in 1.2% (19/1581) of dog faecal samples collected from urban parks across Australia, with reported apparent prevalence of *Strongyloides* spp. in canine faecal samples collected from temperate zones to be 2/669 (0.3%), 1/685 (0.1%) in sub-tropical regions and 16/227 (7%) in tropical regions (Massetti et al., 2022). The prevalence of *S. stercoralis* in dogs has been established to be relatively high in tropical regions, especially in remote communities of Northern and Central Australia, with another study reporting a prevalence of 21.9% (60/274) (Beknazarova et al., 2020). However, the occurrence in temperate regions was limited to case studies (Chapman et al., 2024; Kirkwood and Šlapeta, 2024; Chen et al., 2025).

The detection of *S. stercoralis* infection in a puppy is consistent with global observations that younger animals are more likely to be infected (Basso et al., 2019; Colella et al., 2024). Local *S. stercoralis* exposure from contaminated environments, such as urban parks, is likely rare in temperate areas (Massetti et al., 2022). The positive canine faecal DNA sample was collected in 2020, coinciding with a period of increased pet mobility due to a spike in pet ownership (partially linked to COVID-19), and relocation of dogs from endemic regions in northern Australia to traditionally non-endemic metropolitan areas such as Sydney and Melbourne (Animal Medicines Australia, 2021). Dog relocation has already been suggested as an important risk factor in Europe, facilitating sporadic introductions of *S. stercoralis* into temperate areas (Schnyder et al., 2022). Similar trends were observed in humans across Australia, where the geographic distribution of *S. stercoralis* infection is influenced by travel and migration from endemic regions (Shield et al., 2021). A nationwide seroprevalence study of 81 777 individuals between 2012 and 2016 demonstrated widespread distribution of *S. stercoralis* across Australia, with the highest prevalence among immigrants, travellers and Aboriginal and Torres Strait Islander communities (Shield et al., 2021). These human infection trends may provide valuable insights into canine transmission dynamics, given the comparatively limited diagnostic testing (e.g. Baermann test or PCR) and data available in dogs. Understanding such patterns is essential to inform targeted surveillance strategies, including focused screening of dogs with travel history or those rehomed from endemic areas, to improve early detection and management of *S. stercoralis* in non-endemic regions. Although the concerns regarding emerging cases of *S. stercoralis* infections in dogs from temperate regions may not be as serious as previously suspected, there remains a need for greater efforts in the development of easily accessible and accurate diagnostic testing. The *Strongyloides* qPCR developed by Verweij et al. (2009) was further validated by comparison to a reference Harada–Mori culture technique from 6 g of human faeces, and shown to be 100% concordant, if there were >1 *S. stercoralis* L3 g^−1^ of faeces, while for those with <1 *S. stercoralis* L3 g^−1^ of faeces were only 15% concordant qPCR samples compared to culture (Sultana et al., 2013). Our positive sample was quantified to have >10 *S. stercoralis* L3 g^−1^ of faeces, well within capacity of the qPCR. Sultana et al. (2013) showed that the LOD was 10^−2^ of *S. stercoralis* L3. In our implementation, 1 *S. stercoralis* L3 produced Ct-value of 28.37 (equivalent to ∼2000 target copies of the template), and therefore 100-fold dilutions would theoretically result in Ct-value ∼35 or 20 copies of the template. Culture techniques, such as Harada–Mori culture, that utilize several grams of faeces increase the probability of detection of the *Strongyloides* larvae but are only available in specialized laboratories and take 7 days to produce a result. The Baermann test is also suitable for detecting *Strongyloides* larvae in veterinary settings and diagnostic laboratories, with results available within a day, although its sensitivity is lower than that of PCR (Iatta et al., 2019). PCR remains an accessible, practical and relatively rapid diagnostic tool; the limitation is the increased false negative results in low burden samples because DNA is isolated from only ∼250 mg of faeces. An inherent limitation exists with the *Strongyloides* qPCR developed by Verweij et al. (2009). The assay amplifies DNA from other *Strongyloides* species despite being designed for species-specific detection. Therefore, confirmation using alternative assay and typing is warranted to verify whether the positive sample identified in this study truly represented *S. stercoralis* or another closely related species, such as *S. ratti*, a nematode commonly found in wild rats (Verweij et al., 2009; Sultana et al., 2013; Cole et al., 2023). Cross-contamination through ingestion of rat faeces remains a plausible explanation.

Inclusion of *S. stercoralis* in broader molecular diagnostic panels could enhance surveillance and improve early detection in veterinary settings. Currently, major veterinary diagnostic laboratories do not offer a specific PCR test for *S. stercoralis*. Ideally, both coproscopic and molecular analyses should be performed concurrently to ensure accurate and species-specific diagnosis of *S. stercoralis* (Buonfrate et al., 2018; Iatta et al., 2019; Chan and Thaenkham, 2023). In addition, repeated testing following initial diagnosis and treatment is recommended, as persistence of even a single parasitic female can lead to recrudescence (Iatta et al., 2019; Paradies et al., 2019; Chan and Thaenkham, 2023).

## Conclusion

This study confirms that veterinarians can encounter *S. stercoralis* in dogs living in temperate climates, as demonstrated through a systematic retrospective analysis of archival DNA. However, our findings indicate that the parasite is, in fact ‘*not so slipping under the diagnostic radar*’ of veterinary clinicians in temperate zones, contrary to our initial concerns based on recent reports (Massetti et al., 2022; Chapman et al., 2024; Kirkwood and Šlapeta, 2024; Chen et al., 2025). Currently, the occurrence *S. stercoralis* remains rare in temperate climates in Australia. The *Strongyloides*-qPCR assay showed excellent analytical sensitivity, supporting its adoption by veterinary diagnostic laboratories. The temporal emergence of *S. stercoralis* in temperate regions is likely facilitated by increased dog mobility, including purchase or adoption from endemic areas. Incorporating *S. stercoralis* into routine diagnostic PCR panels and implementing targeted molecular surveillance will be critical for improving early detection in primary care veterinary clinics.

## Data Availability

Sequence data are deposited in GenBank, Short Read Archive accession number: PRJNA1364318 (https://www.ncbi.nlm.nih.gov/bioproject/PRJNA1364318), and associated analyses at LabArchives: https://dx.doi.org/10.25833/e3f3-xc57.

## Table 1 Long description

The table provides information on amplification primers used for the Strongyloides stercoralis 18S rRNA gene assay, which targets a 101 base pair region. It includes two primers, S1221_Stro18S-1530F and S1222_Stro18S-1630R, with their respective sequences. Additionally, the probe primer S1220_Stro18S-2586T is modified with a fluorescent dye and quencher, enhancing detection capabilities. The use of these primers is crucial for accurate amplification and detection of the target gene, with the probe primer's modifications playing a key role in signal clarity. The table does not provide comparative data or trends but focuses on the specific primer sequences and their functional modifications.

Navigate back to Table 1.

## Figure 1 Long description

The image A shows a bar graph illustrating the number of canine faecal samples collected from 2014 to 2024. The x-axis is labeled 'Year' and the y-axis is labeled 'Number of samples'. The graph differentiates samples from two hospitals: University Veterinary Teaching Hospital Camden (UVTHC) and University Veterinary Teaching Hospital Sydney (UVTHS). The bars are stacked, with UVTHC samples in one color and UVTHS samples in another. The highest number of samples was collected in 2019. The image B shows a scatter plot of Ct values from 2014 to 2024. The x-axis is labeled 'Year' and the y-axis is labeled 'Ct value'. The plot includes two categories: 'Above +3 SD' and 'Normal'. A dashed line indicates the mean Ct value of 15.33 and another dashed line shows the mean plus three standard deviations at 25.74. Points above this threshold are marked differently from normal values.

Navigate back to Figure 1.

## Figure 2 Long description

The image A shows a graph with the x-axis labeled 'Real-time PCR cycle - Strongyloides stercoralis 18S rDNA' and the y-axis labeled 'Relative fluorescence units (RFU)'. It displays multiple amplification curves, with a horizontal line indicating the threshold. Two curves are labeled '5x L3' and '1x L3' and a red curve represents a dog sample. The image B shows a graph with the x-axis labeled 'Log starting quantity of Strongyloides stercoralis 18S rDNA' and the y-axis labeled 'Quantification cycle (Cq)'. It features a standard curve with data points marked by circles and a red cross for the dog sample. The graph includes annotations: 'E= 98.9 percent', 'R squared equals 0.999', 'Slope equals 3.348' and 'y-intercept equals 38.765'.

Navigate back to Figure 2.

## Figure 3 Long description

The image A shows a proportional symbol map of canine faecal samples per postcode across Greater Sydney, NSW. An inset highlights the region of Australia included in the study. The image B displays mean centres and directional ellipses of sample distributions submitted to Sydney (UVTHS) and Camden (UVTHC) hospitals, with locations marked as point dots. The image C is a choropleth map showing the number of samples per postcode submitted to Sydney (UVTHS) hospital, with varying intensities. The image D is a choropleth map depicting the number of samples per postcode submitted to Camden (UVTHC) hospital, also with varying intensities. Each map includes a scale and directional arrow for reference.

Navigate back to Figure 3.
